# Detection, transmission and spread of airborne avian influenza and Newcastle disease viruses: experimental and field investigations

**DOI:** 10.1186/s13567-025-01533-9

**Published:** 2025-05-19

**Authors:** Pierre Hostyn, Mieke Steensels, Bénédicte Lambrecht

**Affiliations:** Sciensano, Service of Avian Virology and Immunology, 1180 Brussels, Belgium

**Keywords:** HPAIV, NDV, aerosols, chickens, diagnostic, surveillance, dispersion, environment

## Abstract

Both avian influenza (AI) and Newcastle disease (ND) viruses cause highly contagious respiratory diseases in chicken. These viruses are transmitted through the oro-faecal route, with airborne transmission via virus-laden droplets or dust. In this study, the Coriolis^®^ µ air sampler was evaluated for its suitability to assess the air detection and dispersion of highly pathogenic avian influenza virus (HPAIV) or live Newcastle disease virus (NDV) vaccines between chickens in both experimental and field settings. Experimental assays demonstrated HPAIV and NDV detection in air samples, indicating aerial persistence beyond the end of viral shedding measured in tracheal and cloacal swabs. Viral particles were detected in field air samples taken inside and outside HPAIV H5N1 outbreak farms, with outside aerial dispersion reaching up to 40 m from the exhaust fans. In accordance with these findings, viral particles were detected in air samples both indoors and outdoors from three live NDV-vaccinated farms; however, their aerial dispersion extended only up to 5 m from the exhaust fans. As observed in the NDV controlled assays, high levels of viral concentrations persisted in the air samples, whereas the viral concentrations in the individual swabs collected from the chickens were lower in the live NDV-vaccinated farms. For both the HPAIV and NDV field data, chicken density seemed to impact the viral air concentrations within and outside the studied farms. Coriolis^®^ µ proved effective as a non-invasive method for diagnosing AIV and NDV in both experimental and field studies, highlighting the value of air samples for monitoring poultry disease outbreaks.

**Supplementary Information:**

The online version contains supplementary material available at 10.1186/s13567-025-01533-9.

## Introduction

Avian influenza viruses (AIV) and Newcastle disease viruses (NDV) are members of the families *Orthomyxoviridae* and *Paramyxoviridae*, respectively. Notifiable AIV and NDV are caused by highly pathogenic H5 and H7 subtype AIV strains (HPAIV) [1, 2] and by velogenic NDV strains classified within *Avian orthoavulavirus 1* (vAOAV-1) [1, 3], respectively. Significant morbidity and mortality in Galliformes are induced by both HPAIV and vAOAV-1, leading to substantial economic losses in poultry production [4, 5]. These viruses have a broad spectrum of susceptible avian hosts and circulate within wild bird populations [5–7]. Since 2016, Europe has experienced successive introductions of highly pathogenic (HP) H5Nx influenza viruses of clade 2.3.4.4b [8, 9]. These introductions have resulted in the most devastating HPAIV epizootic in Europe to date, spanning from October 2021 to 2023 [4, 6, 9]. In 2018, a vAOAV-1 epizootic caused by genotype VII.2 emerged in northwestern Europe, including Belgium, primarily affecting backyard birds and some commercial poultry [10]. Since 2020, vAOAV-1 genotype VII.2 outbreaks have been reported in Bangladesh, and the virus has also been isolated in Iran, Israel, and Zambia [11]. The virus has spread from endemic regions to new areas, resulting in localized epidemics [11]. Systemic infections, including respiratory, enteric, and neurotropic tropisms, have been described in chickens for both clade 2.3.4.4b HPH5 and vAOAV-1 infections [12–16]. Owing to these viral tropisms, the Belgian National Reference Laboratory (NRL) uses tracheal, cloacal and brain samples for diagnosing these two notifiable diseases in cases of suspected poultry mortality [1, 17]. In addition to tracheal and cloacal shedding, viral presence in the feather pulp [10, 18] and epidermal cells [18] has been observed after 2.3.4.4b HPH5 and vAOAV-1 infections in chickens. The presence of viruses in these excretions and desquamations suggests that these viruses could be detected in the aerial environment.

Air sampling has previously been evaluated for the detection and spread of pathogenic respiratory viruses such as SARS-CoV-2 in humans [19] and AIV in birds [20]. In this study, Coriolis^®^ µ air sampling was assessed as an alternative to individual bird sampling for AIV and NDV diagnosis, with a focus on detection at the population level rather than at the individual level. Previous studies have reported the detection of both low pathogenic avian influenza virus (LPAIV) and HPAIV strains, including the H9N2, H7N9 and H5N6 subtypes, in Chinese live markets using the Coriolis^®^ µ sampler [21]. The use of Coriolis^®^ µ was not previously described for the identification of NDV viral particles.

Implementing this non-invasive method in the field would prevent the manipulation of contaminated animals, as it is cost-saving and easier to apply in various settings. The current study included both laboratory-controlled assays and field evaluations of air samples. In addition to diagnostic purposes, the assessment of air sampling for viral detection and dispersion around HPAIV- and NDV-infected farms was performed.

## Materials and methods

### Viral strains

HPAIV experimental infections were conducted with clade 2.3.4.4b HPH5N8, A/Anser_albifrons/Belgium/11956_005/2020 (BE-HPH5N8_2020, EPI_ISL_661313 GISAID) strains isolated from wild geese in Belgium by the National Reference Laboratory for Avian Influenza (NRL AI). The experimental live NDV vaccination was carried out with a vaccine strain, LaSota, which was isolated from Belgian diagnostic field samples by the National Reference Laboratory for Newcastle Disease (NRL ND). Viral stocks were produced through amplification following inoculation into the allantoic cavity of 9-day-old embryonated specific pathogen-free (SPF) eggs, which were subsequently incubated at 37 °C. The viral titres were determined by the standard protocol [22], and the stocks were stored at −70 °C for later use.

### Animals and housing

The in vivo experiments were performed using in-house hatched SPF White Leghorn chickens (Lohmann VALO Biedia™, Germany). The chicks were housed directly in biosafety level 3 (BSL-3) isolators (Montair©-HM1500, The Netherlands) with food and water ad libitum until the end of the experiments.

### Monitoring of clinical signs

Daily monitoring was conducted on the birds until the end of the experiments. Clinical scores of 0, 1, 2 and 3 correspond to “no sickness”, “sick”, “severely sick” and “death”, respectively. If one of the following symptoms was observed: respiratory involvement, depression, diarrhoea, cyanosis of the exposed skin or wattles, red eyes, facial oedema, or nervous signs, the bird was considered as “sick” and was assigned a score of 1. If two or more of these symptoms were observed, the animals were categorized as “severely sick” with a score of 2 [23]. Human endpoints were applied if lethargy, accompanied by a lack of feeding and hydration for more than 24 h, was observed in the animals. At the end of the experiment, all surviving animals were euthanized by exsanguination under anaesthesia.

### Air sampling

Air samples were collected in 15 mL of collection mixture composed of 0.005% Triton X-100 diluted in water using the Coriolis^®^ µ air sampler (Bertin^®^, Montigny-le-Bretonneux, France). The air collection cycle is defined by the suction flow rate and duration, with adaptations between the BSL-3 controlled (250 L/min for 4 min, the entire isolator volume of 1.05 m^3^) and field assays (300 L/min for 10 min, 3 m^3^).

For the experimental studies, all the sampling equipment was disinfected after each sampling day. The flowing cane and air intake were submerged in a Sekusept™ (Ecolab^®^, Groot Bijgaarden, Belgium) disinfectant bath for one hour while exposed to UV light. After disinfection, these components were thoroughly rinsed and autoclaved before being used for subsequent sampling. The Coriolis device, which contains the electronics, was disinfected twice using Virocid^®^ RTU spray (CID LINES^®^, Ieper, Belgium), followed by one hour of UV light exposure.

For the field studies, two Coriolis^®^ µ devices, three flowing canes, and three air intakes were used. One Coriolis^®^ µ device was sampled at greater distances (e.g., for Outbreak II: 40 m and 20 m), whereas the other device was sampled at shorter distances (e.g., for Outbreak II: 5 m and 0 m). To minimize the risk of contamination outside the barns, sampling was performed in the areas farthest from the barns and continued in the areas closest to the barns. Inside the barn, the same Coriolis^®^ µ used for sampling the farthest distance was employed, but with a newly sterilized flowing cane and air intake to prevent cross-contamination. After each field sampling session, the disinfection procedure described for the experimental setup was repeated to ensure sterile conditions for subsequent sampling.

### Experimental setup

Approval for each animal experiment was granted by the Sciensano Biosecurity and Ethics Commission, with authorization numbers 20211013-02 and 20180222-02, respectively, in accordance with national and European regulations.

#### HPH5Nx infection

Thirteen 5-week-old chickens were exposed to the BE-HPH5N8_2020 strain, 3 of which were infected oculonasally using a 10^4.5^ EID_50_/100 µL infectious dose. At 6 h post infection, the 10 contact birds were added to the 3 infected individuals. At 2, 5, 7, 9, 14, and 21 days post infection (dpi), tracheal and cloacal swabs and air samples were collected during the experiments. Tracheal and cloacal swabs were hydrated in brain‒heart infusion (BHI) medium supplemented with antibiotics (10^7^ U/L penicillin, 2 g/L streptomycin, 1 g/L gentamycin sulfate and 650 mg/L kanamycin sulfate) and stored at −70 °C until analysis. The tracheal and cloacal sampling was stopped when all the animals had died at 6 dpi.

#### Live NDV vaccination

The LaSota vaccine strain was inoculated into 42 two-week-old SPF chickens using a 10^6^ EID_50_/100 µL dose. At 6, 8, 10, and 15 days post-vaccination (dpv), tracheal and cloacal swabs were taken from 5 randomly selected individuals within the BSL-3 isolator. At these timepoints, air samples were simultaneously collected, as previously described.

### Field assay setup

#### HPH5Nx outbreaks

Air sampling was conducted both before culling and before the repopulation of two HPH5N1 outbreak sites that were declared positive in December 2021; these sites were identified as Outbreaks I and II. In Outbreak I, 28 000 broiler chickens were housed on the ground, leading to a total population density of 17 individuals per m^2^. In Outbreak II, 162 000 layer chickens were housed in battery cages at different levels, leading to a total population density of 45 individuals per m^2^. Samples were collected inside and outside the farms on either side of the air expulsion system, as well as at distances of 5, 20, 40 and 50 m from the air exhaust system. Additionally, environmental swabs of wet grass and a water puddle were collected in front of the air exhaust system of Outbreak II.

#### Live NDV vaccination

This study was conducted concurrently with live NDV Avishield^®^ ND (Genera Inc., Kalinovica, Croatia) spray vaccination, using 1000 doses of the vaccine in 150–300 mL of distilled water, with droplet sizes averaging between 115 and 240 µm. At 4, 7 and 14 dpv, air samples and individual swabs were collected from three farms: Farm I, Farm II and Farm III. In Farm I, 54 650 layer chickens were housed on the ground, leading to a total population density of 30 individuals per m^2^. In Farm II, 52 250 layer chickens were housed on the ground, leading to a total population density of 37 individuals per m^2^. In Farm III, 51 450 layer chickens were housed on the ground, leading to a total population density of 42 individuals per m^2^. At each time point, 10 pools of tracheal and cloacal swabs were collected, including 5 pools of tracheal swabs and 5 pools of cloacal swabs. Tracheal or cloacal swabs from 5 randomly selected individuals within the farms were included in each pool. Simultaneously, with individual sampling, air samples were collected inside and outside the farms on either side of the air expulsion system, as well as at distances of 5, 25 and 50 m from the air exhaust system. Additionally, dust swabs were collected only outside, in front of the air exhaust system of each farm, at different time points.

### Detection and quantification of viral RNA by RRT‒PCR

The collected air samples were concentrated prior to extraction with Amicon^®^ Ultra15 Centrifugal Filters (Merck Life Science BV, Overijse, Belgium) at 4000 rotations per minute (RPM) for 40 min. Viral RNA from the air samples was extracted using the High Pure Viral Nucleic Acid Kit (Roche, Machelen, Belgium). Viral RNA from individual swabs was extracted via the MagMax^®^-AI/ND 96 viral RNA kit (Ambion-Applied Biosystems, Lennik, Belgium). RNA was extracted from 200 µL of concentrated air samples or BHI-immersed swabs. Purified RNA was eluted in a final volume of 50 µL.

Viral detection, following the laboratory accredited procedure, which is based on the protocol described by Spackman et al. [24] and Wise et al*.* [25], was performed via real-time reverse transcriptase‒polymerase chain reaction (RRT‒PCR), which targets the conserved region of the M gene for AIV and NDV. Briefly, RRT-PCR was performed on 2 µL of purified RNA using the AgPath-ID^®^ One-Step Kit (Life Technologies, Merelbeke, Belgium). The RRT-PCR amplification was conducted on a LightCycler^®^ 480 System (Roche, Machelen, Belgium), with 1 cycle of reverse transcription at 50 °C for 30 min, followed by 1 cycle of denaturation at 95 °C for 10 min and 50 PCR amplification cycles (15 s at 95 °C, 34 s at 54 °C and 10 s at 72 °C). The standard curve used for AIV relative quantification was based on synthetic M-gene RNA, which was derived from a dilution of a LPAIV H5N1 virus stock (“A/Swan/Hungary/4571/2006”) titrated on eggs. The standard curve used for NDV relative quantification was based on synthetic M-gene RNA, which was derived from a dilution of a vAOAV-1 virus stock (“998/2011”) titrated on eggs. The Cp cut-off values for AIV and NDV were set at 40 and 38 cycles, and 95% detection limits of 10^2.7^ and 10^3.85^ viral RNA genome copies per mL were established, respectively. Viral concentrations are expressed as the log_10_ value of the number of viral RNA genome copies per mL of swab BHI or air sample collection mixture: log_10_ (RNA copies/mL). For statistical evaluation, a value of 1 log_10_ (RNA copies/mL) was assigned to negative samples.

### Statistical analyses

Statistical analyses were conducted with GraphPad Prism 9.4.1 software, and significance was determined at *p* < 0.05. The raw data were subjected to nonparametric Wilcoxon signed-rank tests and Mann‒Whitney U tests to compare two groups with dependent or independent samples, respectively.

## Results

### Virus detection in individual swabs and air under BSL-3-controlled assays

**Figure 1:**
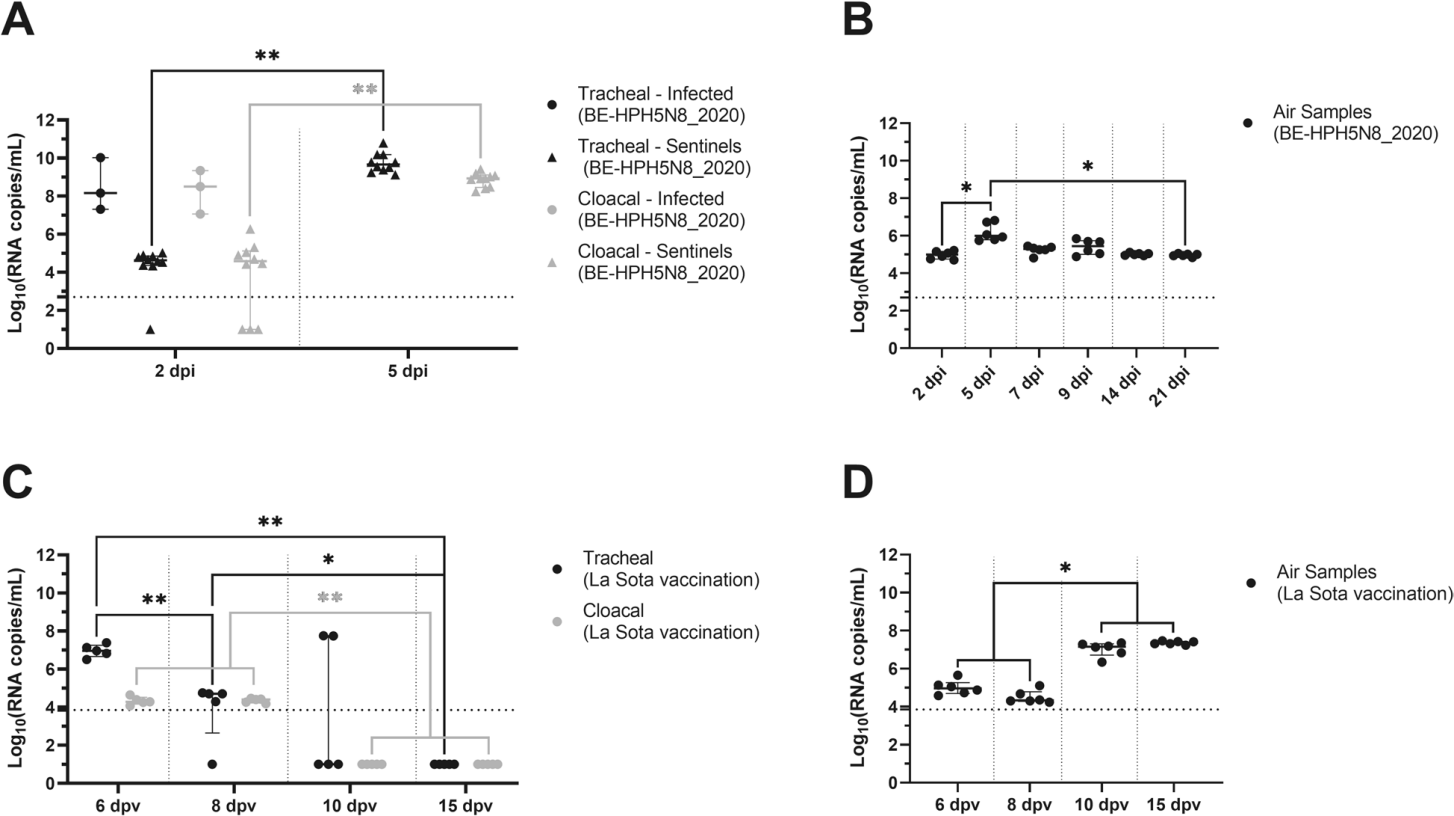
**Air sampling and individual swabbing under controlled BSL-3 experimental settings.** **A** Viral concentrations detected in tracheal and cloacal swabs. **B** Air samples from chickens exposed to the BE-HPH5N8_2020 strain. **C** Viral concentrations detected in tracheal and cloacal swabs. **D** Air samples from chickens vaccinated with the LaSota strain. The detected viral concentrations are expressed as log_10_ values (RNA copies/mL). For BE-HPH5N8_2020 infection, air data were obtained at different sampling times: 2, 5, 7, 9, 14 and 21 dpi. For live NDV vaccination, data are presented for different sampling times of 6, 8, 10 and 15 dpv. The nonparametric Wilcoxon signed-rank test was used to compare the viral loads excreted between each timepoint for air sampling and individual swabs for BE-HPH5N8_2020 infection (ns: *p* > 0.05; *: *p* < 0.05; **: *p* < 0.01—Wilcoxon signed-rank test). The nonparametric Mann‒Whitney U test was used to compare the viral loads excreted between each timepoint for individual swabs for live NDV vaccination (ns: *p* > 0.05; *: *p* < 0.05; **: *p* < 0.01—Mann‒Whitney U test).

Among the BE-HPH5N8_2020-infected birds, one died at 2 dpi, two at 3 dpi, while the contact birds showed a later mortality pattern: nine died at 5 dpi, and the last died at 6 dpi. Before death, all individuals involved in the experiment displayed severe avian influenza symptoms, including respiratory distress, depression, diarrhoea, cyanosis of the skin or wattles, red eyes, facial swelling, and neurological signs. In tracheal and cloacal swabs from BE-HPH5N8_2020-infected birds, viral particles were detected only before death at 2 dpi. Median viral titres of 8.17 and 8.50 log_10_ (RNA copies/mL) were detected in tracheal and cloacal swabs, respectively (Figure 1A). At 5 dpi, both tracheal shedding and cloacal shedding were significantly greater in sentinel birds exposed to BE-HPH5N8_2020 than in those at 2 dpi (Wilcoxon signed-rank test, *p* = 0.002). The median viral titres at 5 dpi were 9.76 and 8.93 log_10_ (RNA copies/mL), respectively (Figure 1A), exceeding the viral excretion levels of the initially infected birds.

Following live NDV vaccination, a progressive decrease in tracheal shedding was observed between 6 and 10 dpv before complete extinction at 15 dpv. A significant decrease in median viral titres for tracheal shedding was noted from 6 to 8 dpv (Mann‒Whitney U test, *p* = 0.0079) and from 8 to 15 dpv (Mann‒Whitney U test, *p* = 0.0476). Median viral titres of 6.97, 4.71, 1 and 1 log_10_ (RNA copies/mL) were detected in tracheal swabs at 6, 8, 10 and 15 dpv, respectively. Cloacal viral excretion was minimal at 6 and 8 dpi, with a significant decrease from 8 to 10 dpv (Mann‒Whitney U test, *p* = 0.0079) (Figure 1C). Median viral titres of 4.28, 4.41, 1 and 1 log_10_ (RNA copies/mL) were detected in cloacal swabs at 6, 8, 10 and 15 dpv, respectively (Figure 1C).

Viral particles were detected in air samples collected at 2 dpi from the BSL-3 isolator containing BE-HPH5N8_2020-infected chickens (Figure 1B) and at 6 dpv for the LaSota-vaccinated chickens (Figure 1D). The peak viral concentrations were subsequently observed at 5 dpi for BE-HPH5N8_2020 (Figure 1B) and at 15 dpv for LaSota (Figure 1D). At these peaks, median viral titres of 6.00 and 7.37 log_10_ (RNA copies/mL) were detected. For BE-HPH5N8_2020, a significant increase in viral concentrations in air samples was observed from 2 to 5 dpi (Wilcoxon signed-rank test, *p* = 0.0313), followed by a gradual decrease, which was significantly lower at 21 dpi than at 5 dpi (Wilcoxon signed-rank test, *p* = 0.0313) (Figure 1B). A significant increase in the viral concentrations of LaSota in air samples was observed at both 10 and 15 dpv compared with those at 6 and 8 dpv (Wilcoxon signed-rank test, *p* = 0.0313) (Figure 1D).

In the absence of tracheal and cloacal shedding, the air samples remained positive, with median viral titres of 4.97 log_10_ (RNA copies/mL) at 21 dpi and 7.37 log_10_ (RNA copies/mL) at 15 dpv from BSL-3 isolator-containing BE-HPH5N8_2020-infected chickens (Figures 1A and B) and LaSota-vaccinated chickens (Figures 1C and D), respectively. For HPAIV, this aerial persistence was confirmed in additional experimental assays with a Belgian clade 2.3.4.4b H5N8 virus isolated in 2017 (BE-HPH5N8_2017) (Additional file 1).

### Aerial viral detection within HPH5N1-infected farms and the surrounding viral dispersion

In December 2021, air samples were collected prior to culling at two HPH5N1-infected farms, a broiler farm (Outbreak I, chicken density 17/m^2^) and a layer farm (Outbreak II, chicken density of 45/m^2^). The HPH5N1-positive status of these two farms was officially confirmed by RRT-PCR targeting the M, H5 and N1 genes, with Cp values of 21.01, 20.87, and 22.97 for Outbreak I and 21.88, 20.32, and 23.82 for Outbreak II detected in brain homogenates (Belgian NRL AI/ND values; data not shown). Sequencing confirmed the highly pathogenic pathotype of the two outbreaks through the identification of a polybasic cleavage site (PLREKRRKRGLF).

**Figure 2:**
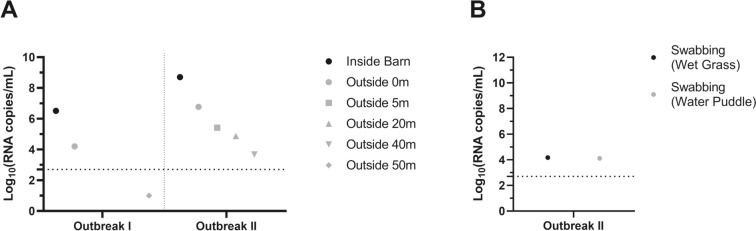
**Air sampling and environmental swabbing of clade 2.3.4.4b HPH5N1 outbreak sites.** **A** Viral concentrations detected in air samples (**B**) and environmental swabbing of wet grass and a water puddle at two HPH5N1 outbreak sites (Outbreaks I and II). Viral concentrations were expressed as log_10_ (RNA copies/mL) values for both outbreaks. Environmental swabs were only collected during outbreak II in front of the air exhaust system.

In Outbreak I, a reduction in the viral concentration was observed between the air samples inside and outside each side of the air exhaust system, with viral titres of 7.41 and 4.20 log_10_ (RNA copies/mL), respectively (Figure 2A). No viral particles were detected in the single sample taken outside at a distance of 50 m from the air exhaust system. In Outbreak II, viral concentrations of 9.48 and 7.49 log_10_ (RNA copies/mL) were detected on both sides of the air exhaust system. Similar to Outbreak I, a reduction in the viral concentration was observed between samples taken on either side of the air exhaust system (Figure 2A). Viral concentrations of 5.42, 4.88 and 3.68 log_10_ (RNA copies/mL) were detected in air samples taken 5, 20 and 40 m from the exhaust system, respectively (Figure 2A). A greater airborne presence was observed both inside and outside of Outbreak II than outside of Outbreak I (Figure 2A). Viral isolation of embryonated eggs was successful only from the indoor air sample from Outbreak II.

Environmental swabs were taken from wet grass and a water puddle near the air exhaust system of Outbreak II. Similar viral concentrations were observed in both samples, with viral titres of 4.18 and 4.11 log_10_ (RNA copies/mL), respectively (Figure 2B). After culling, disinfection, and a three-week sanitary vacancy period, air samples were collected from both sides of the air exhaust, just before the repopulation of both farms. These samples confirmed the pathogen-free status of the former outbreak sites.

### Aerial viral detection within live NDV-vaccinated farms and the surrounding viral dispersion

Viral particles were successfully detected in air samples taken at different timepoints after vaccination, both inside and outside of three live NDV spray-vaccinated farms referred to as Farm I, Farm II and Farm III, with population densities of 30, 37 and 42 chickens per m^2^, respectively. Similar viral concentrations were observed in the air samples collected from each side of the air exhaust system on all three farms at 4 and 7 dpv, with viral concentrations ranging from 4.44 to 5.92 log_10_ (RNA copies/mL). At 14 dpv, viral particles were detected inside Farm II and Farm III, whereas viral particles were detected outside Farm II (Table 1). An outside viral dispersion was observed in air samples taken 5 m from the exhaust system for Farm I and Farm III at 4 dpv. No viral particles were detected in air samples taken at 25 and 50 m from the air exhaust system at any of the farms at any given timepoint (Table 1). Viral isolation from embryonated eggs was unsuccessful for all the air samples from the three farms.

**Table 1:** **Summary of viral detection within live NDV vaccinated farms**

		Viral titre Log_10_ (RNA copies/mL)								
		Farm I			Farm II			Farm III		
		4 dpv	7 dpv	14 dpv	4 Dpv	7 dpv	14 dpv	4 Dpv	7 dpv	14 dpv
Air sampling	Inside	4.88	5.05	1	4.44	5.01	4.71	5.48	5.31	4.96
	Outside 0 m	5.92	5.37	1	4.52	5.23	4.57	4.76	5.33	1
	Outside 5 m	5.25	1	1	1	1	1	4.92	1	1
	Outside 25 m	1	1	1	1	1	1	1	1	1
	Outside 50 m	1	1	1	1	1	1	1	1	1
Swabbing	Tracheal swabs (Pos./Total)	1 [4.64] (2/5)	1 [3.08] (1/5)	1 (0/5)	6.15 [2.96] (4/5)	6.04 [4.14] (4/5)	1 [1.95] (1/5)	6.82 [4.77] (4/5)	6.04 [6.20] (3/5)	1 [2.67] (1/5)
	Cloacal swabs (Pos./Total)	1 (0/5)	1 (0/5)	1 (0/5)	1 (0/5)	1 (0/5)	1 (0/5)	1 (0/5)	1 (0/5)	1 (0/5)
	Dust swabs	1	1	1	4.83	4.51	4.50	4.91	4.95	1

Viral titres were expressed as Log_10_ (RNA copies/mL) for all farms at each timepoints and for each sampling of the study. For tracheal and cloacal swabs, the number of positives swabs pool out of the total were depicted in this table (Pos./Total). Interquartile ranges [IQR] were specified for the median viral concentration of positives tracheal and cloacal swabs. The nonparametric Mann‑Whitney U test was performed to compare viral concentrations detected in sheddings between the different timepoints of a same farm (Farm I, Farm II or Farm III) (ns: *p* > 0.05; *: *p* < 0.05; *: *p* < 0.01—Mann‑Whitney U test).

After live NDV Avishield^®^ ND vaccination, no viral particles were detected in cloacal pooled swabs taken from any of the three farms (Table 1). Over time, a progressive decrease in tracheal shedding was observed on all three farms. At 4 dpv, 40%, 80%, and 80% of the pooled tracheal swabs were positive for Farm I, Farm II, and Farm III, respectively. By 14 dpv, these percentages decreased to 0%, 20%, and 20% for Farm I, Farm II, and Farm III, respectively. For Farm II, a significant decrease in tracheal shedding was observed between 7 and 14 dpv (Mann–Whitney U test, *p* = 0.0476) (Table 1).

Dust swabs were also collected from outside the exhaust system, where viral particles were detected in dust from Farm II and Farm III at 4 and 7 dpv. Additionally, at 14 dpv, the virus was detected in dust swabs from Farm II, with viral concentrations ranging from 4.50 to 4.95 log_10_ (RNA copies/mL) in the positive dust swabs (Table 1).

## Discussion

Over the past decade, both clade 2.3.4.4 HPAIV and genotype VII.2 vAOAV-1 strains have affected Europe and Belgium, resulting in substantial economic losses in poultry [8, 10]. These highly contagious diseases in birds must be noted to competent authorities. In this context, it is essential to implement surveillance tools for early detection of HPAIV and vAOAV-1 outbreaks, enabling rapid culling and disinfection measures to prevent virus spread. This study evaluated the effectiveness of the Coriolis^®^ µ sampler as a non-invasive and cost-effective method for monitoring disease and compared it with the reference method based on individual bird sampling. Our study specifically investigated the suitability of air sampling for the identification of circulating clade 2.3.4.4b HPAIV strains. In contrast, owing to the rare occurrence of vAOAV-1 infections in poultry, live attenuated NDV vaccines were used as surrogates for the controlled assay and field evaluation. The findings of the present study enhance the understanding of the presence and dispersion of viruses in the air, complementing the literature on airborne virus sampling [26].

In this study, the use of a Coriolis^®^ µ cyclonic air sampler successfully led to the detection of HPAIV and NDV in air samples from both controlled and field settings. AIV and NDV detection in air samples has been previously described using various air sampling technologies, including filter-based, impact, impingement and cyclonic methods [27–32]. Prior studies have reported the detection of AIV in controlled assays [27, 28], at live poultry markets [29] and during HPAIV outbreaks [30] using one or several of these air sampling technologies. Strains of vAOAV-1 have been successfully detected under experimental challenge conditions by using the impingement method [31], and infections under field conditions have been detected in samples collected with filter-based samplers [32]. In addition to AIV and NDV, other avian viruses, such as infectious bursal disease virus (IBDV) and infectious laryngotracheitis virus (ILTV), have been detected via air samplers [33, 34]. Specifically, IBDV was detected under experimental conditions in broiler chickens [33], whereas ILTV was detected in broiler farms [34]. Other avian pathogens, such as *Campylobacter* and *Salmonella typhimurium*, which cause significant bacterial contamination in chicken carcasses [35], have been found in air samples collected from infected flocks and in controlled studies [36].

Data comparing the sensitivity of these air sampling technologies and individual swabbing methods are limited in the current literature. Filaire et al. recently compared the sensitivity of HPAIV detection in individual swabs and air samples using the Coriolis^®^ compact cyclonic air sampler in 63 poultry farms during the 2020–2021 epizootic of clade 2.3.4.4b HPH5N8 in France [37]. This study suggested that the Coriolis^®^ compact sampling method is more sensitive than individual swabbing in flocks without clinical signs [37]. Unlike in Filaire’s study, in our study of controlled BE-HPH5N8_2020 infection, viral particles in air samples and swabs were detected equally in infected chickens, which displayed clinical signs. This observation was confirmed in the additional chicken experimental infection with a Belgian clade 2.3.4.4.b strain isolated in 2017. In both the control and field live NDV vaccination systems, viral particles were detected in both air samples and individual swabs during the early stages of sampling. Under controlled conditions, viral persistence was observed in air samples following infection with HPAIV (BE-HPH5N8_2020) and live NDV vaccination, in contrast to what was observed in individual samples. For the BE-HPH5N8_2020 strain, stable viral levels were detected for up to 15 days after the removal of the last cadaver, as was also observed with the BE-HPH5N8_2017 strain. Similarly, Filaire et al. reported sustained viral levels in air samples, while the levels in individual swab samples decreased in mallard ducks infected with HPH5N8 strains [28]. Although studies in ducks have shown similar results, the patterns of viral shedding differ significantly between these species, greatly limiting the ability to compare studies directly [28]. Further studies in chickens are needed to confirm these observations. In previous experimental NDV infections in chickens with the NX1069 and LaSota strains, virus detection has been compared in cloacal swabs and in air samples up to 40 dpi [38]. Both strains were detected in air samples from 7 to 40 dpi, but cloacal shedding was still detected at 40 dpi in 57% and 64% of individuals infected with the NX1069 and LaSota strains, respectively [38]. Interestingly, the viral concentrations in air samples remained stable, even as the viral concentrations in individual swabs progressively decreased.

For biosafety purposes, air sampling was allowed on both farms affected by an HPAIV outbreak just before culling and after decontamination, limiting the field evaluation of HPAIV persistence in the air. Nevertheless, the detection of the NDV vaccine strain under field conditions demonstrated the persistence of the virus in the air for 7–14 days inside and outside the vaccinated barns, even when the measured degree of tracheal shedding was reduced. Given the similar persistence of HPAIV and NDV observed during our controlled assays, the presence of HPAIV in the air may persist just outside the exhaust fans of HPAIV-infected farms and contribute to the local spread of the virus. These findings highlight the importance of rapid culling and disinfection following the detection of HPAIV and NDV outbreaks to manage airborne viral persistence. The viral analysis of air samples also confirmed the absence of pathogens prior to the outbreak sites, supporting the effectiveness of air sampling in outbreak management and biosecurity monitoring.

During the clade 2.3.4.4b epizootic in France in 2017, viral particles were detected up to 110 m downwind from a chicken farm, while cadavers were loaded onto trucks during outbreak disinfection efforts [39]. Our results revealed that viral spread during clade 2.3.4.4b HPH5N1 outbreaks reached up to 40 m around Outbreak II, with a farm containing 45 birds/m^2^ (162 000 layer chickens). At the site of Outbreak I, whose farm had a lower density of 17 birds/m^2^ (28 000 layer chickens), HPAIV was detected solely near the exhaust system. A higher viral concentration was detected inside the barn of the most densely populated farm (Outbreak II) than inside the barn of the least populated farm (Outbreak I), suggesting that animal density likely influences viral concentrations and dispersion within and around contaminated barns. These findings align with a 2015 study in the USA, which demonstrated that airborne transmission extended 150 m downwind during a clade 2.3.4.4 HPH5N2 outbreak involving 575 000 chickens and reached up to 1000 m in a separate HPH5N2 outbreak with 1.8 million chickens [30]. In a recent study describing a clade 2.3.4.4b HPH5N1 epizootic in the UK, no aerial dispersion was observed around a chicken-infected farm containing 37,500 chickens [40]. Further evaluations are needed to confirm the impact of poultry density on viral dispersion around HPAIV outbreaks.

Few studies have described the airborne dispersion of velogenic NDV strains, with viral particles detected both inside and within one meter of two Californian chicken farms contaminated in 2003 [32]. In that study, air samples were collected in front of the exhaust system, and further distances from the farm were not evaluated. The detection and dissemination of an NDV vaccine strain were evaluated under field conditions on three farms, each of which was vaccinated with the Avishield^®^ ND strain, which has exclusive tracheal tropism with low viral replication [41]. In farms with denser chicken populations (Farm II and Farm III), NDV was detected in air samples up to 14 dpv inside the barn, whereas it was detectable up to 5 m around NDV-vaccinated Farms I and III. Given that vAOAV-1 induces both tracheal and cloacal shedding, the viral concentrations detected within and around vAOAV-1 outbreaks may exceed those observed in this NDV vaccination study. For example, for HPAIV, further evaluations are needed to confirm the impact of poultry density on viral dispersion around NDV-vaccinated farms or during vAOAV-1 outbreaks.

This study also highlights the effectiveness of environmental samples such as dust, grass, and water swabs in detecting HPAIV and NDV viral particles around contaminated barns. This viral deposition around farms infected with clade 2.3.4.4b HPAIV increases the risk of secondary infections in poultry or other livestock and potential spillback to wild bird populations. These findings are supported by the previously reported detection of clade 2.3.4.4b HPH5N1 in dust near chicken farm vents in the UK [40]. Filaire et al. also highlighted the effectiveness of environmental sampling for HPAIV detection by collecting dust from feeders and walls on French duck and chicken farms [37]. Nevertheless, unlike Filaire et al., environmental swabbing, such as dust swabs, was conducted only outside, in front of the air exhaust system, to evaluate viral deposition around HPAI- and NDV-contaminated barns. Additionally, the detection of infectious bronchitis virus (IBV) and NDV particles was recently described in dust samples collected from vaccinated broiler chicken farms [42], suggesting that dust sampling could be a valuable non-invasive method for AIV and NDV surveillance in the future.

Despite promising results, some aspects of using the Coriolis^®^ µ for diagnostic purposes may need further consideration. Indeed, the Coriolis^®^ µ is less user friendly than devices such as the Coriolis^®^ Compact [37] and the AeroCollect^®^ [36]. However, the Coriolis^®^ µ offers a higher maximum flow rate of 300 L/min than does the 50 L/min of the Coriolis^®^ Compact. These differences in flow rate could impact the sensitivity of viral detection, especially in environments with very low viral loads, such as outdoor settings, and warrant further investigation to confirm this hypothesis.

A key technical aspect of this study is the use of water with Triton X-100 as the collection liquid for Coriolis^®^ µ air sampling, as described in the materials and methods section. This choice was made to enhance the detection of low viral concentrations in the field, as previous studies have demonstrated its ability to detect low viral concentrations up to 110 m downwind at HPAI outbreak sites [39]. However, since Triton X-100 is a detergent, it is not ideal for assessing viral viability. Although the virus was successfully isolated from an indoor air sample collected during HPH5N1 Outbreak II, likely due to the high viral concentration, the viability of viral particles in outdoor air samples could not be confirmed. This study described only viral spread by detecting viral RNA outside the barns without confirming viral viability, which should be considered a limitation of the study.

In conclusion, this study highlights the Coriolis^®^ µ air sampler as a potentially effective tool for detecting and monitoring avian respiratory disease outbreaks within poultry flocks. Unlike traditional individual swabbing methods, air sampling methods detect viral particles outside barns infected with HPAIV or vaccinated with live NDV without direct contact with the birds. Viral analysis of air samples confirmed the absence of pathogens before the outbreak sites were repopulated, demonstrating the potential of air samplers for outbreak management and biosecurity. Field experiments suggest that factors such as animal density may affect airborne viral concentrations and persistence, providing insights into airborne transmission dynamics. Environmental samples, including dust, grass and water swabs, have also been shown to be effective in detecting HPAIV and NDV in areas surrounding contaminated barns, supporting the broader use of air sampling in disease monitoring. While promising, further evaluation is necessary to fully integrate air sampling into existing surveillance protocols, which could improve early detection, outbreak control and biosecurity practices for managing avian respiratory disease.

## Supplementary Information

**Additional file 1. Air and individual swabbing following BSL-3 BE-HPH5N8_2017 infection.** (A) Viral concentrations detected in tracheal and cloacal swabs (B) and air samples from chickens exposed to the BE-HPH5N8_2017 strain. The detected viral concentrations are expressed as Log_10_ values (RNA copies/mL). For BE-HPH5N8_2017 infection, air data are presented for different sampling times: 2, 5, 7, 9, 14 and 21 dpi. The nonparametric Wilcoxon signed-rank test was used to compare the viral loads excreted between each timepoint for the same sampling method (air samples, tracheal samples and cloacal samples) (ns: *p* > 0.05; *: *p* < 0.05; **: *p* < 0.01 – Wilcoxon signed-rank test).

## Data Availability

The datasets used and/or analysed during the current study are available from the corresponding author, PH, upon reasonable request.
