# Supplementary figures and images for: Detection, transmission and spread of airborne avian influenza and Newcastle disease viruses: experimental and field investigations
Source: Vet Res. 2025 May 19;56:102. doi: 10.1186/s13567-025-01533-9 (PMC12087104; doi:10.1186/s13567-025-01533-9)

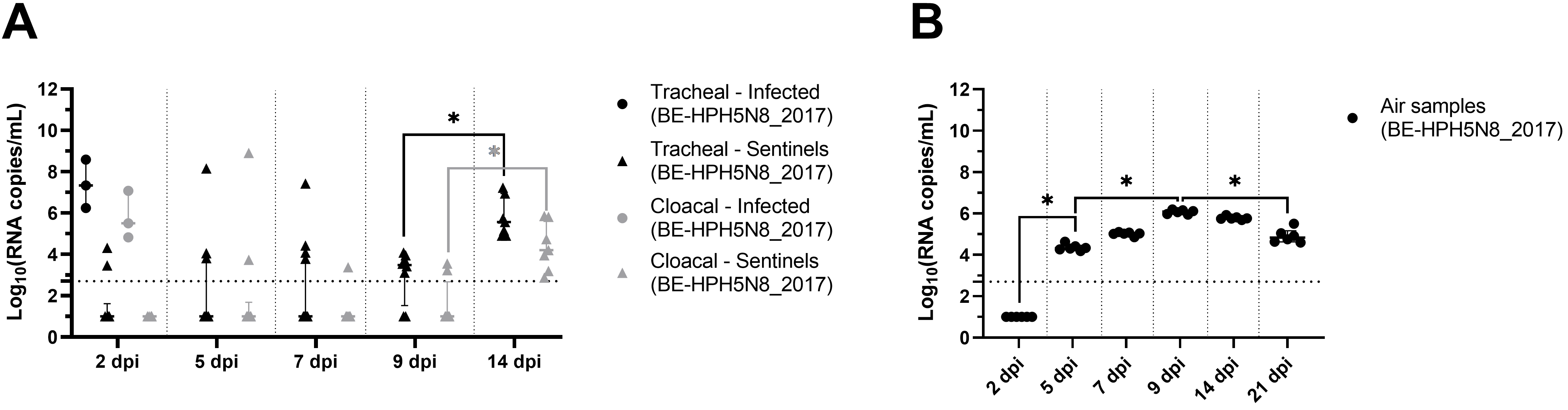

Supplement: Supplementary file 1 — Additional file 1. Air and individual swabbing following BSL-3 BE-HPH5N8_2017 infection. (A) Viral concentrations detected in tracheal and cloacal swabs (B) and air samples from chickens exposed to the BE-HPH5N8_2017 strain. The detected viral concentrations are expressed as Log10 values (RNA copies/mL). For BE-HPH5N8_2017 infection, air data are presented for different sampling times: 2, 5, 7, 9, 14 and 21 dpi. The nonparametric Wilcoxon signed-rank test was used to compare the viral loads excreted between each timepoint for the same sampling method (air samples, tracheal samples and cloacal samples) (ns: p > 0.05; *: p < 0.05; **: p < 0.01 – Wilcoxon signed-rank test). [file 13567_2025_1533_MOESM1_ESM.tif]
